# Hygiene on maternity units: lessons from a needs assessment in Bangladesh and India

**DOI:** 10.3402/gha.v9.32541

**Published:** 2016-12-12

**Authors:** Suzanne Cross, Kaosar Afsana, Morsheda Banu, Dileep Mavalankar, Emma Morrison, Atiya Rahman, Tapash Roy, Deepak Saxena, Kranti Vora, Wendy J Graham

**Affiliations:** 1 The Soapbox Collaborative, Aberdeen, Scotland, UK; 2 Health, Nutrition & Population Programme, BRAC, Dhaka Division, Dhaka, Bangladesh; 3 Research & Evaluation Division, BRAC, Dhaka Division, Dhaka, Bangladesh; 4 Indian Institute of Public Health, Gandhinagar, Ahmedabad, India; 5 Division of Social Research in Medicines and Health, University of Nottingham, Nottingham, UK; 6 Department of Infectious Disease Epidemiology, London School of Hygiene & Tropical Medicine, London, UK

**Keywords:** WASH, infection prevention, maternal and newborn health, environmental hygiene, visual cleanliness

## Abstract

**Background:**

As the proportion of deliveries in health institutions increases in low- and middle-income countries, so do the challenges of maintaining standards of hygiene and preventing healthcare-associated infections (HCAIs) in mothers and babies. Adequate water, sanitation, and hygiene (WASH) and infection prevention and control (IPC) in these settings should be seen as integral parts of the broader domain of quality care. Assessment approaches are needed which capture standards for both WASH and IPC, and so inform quality improvement processes.

**Design:**

A needs assessment was conducted in seven maternity units in Gujarat, India, and eight in Dhaka Division, Bangladesh in 2014. The WASH & CLEAN study developed and applied a suite of tools – a ‘walkthrough checklist’ which included the collection of swab samples, a facility needs assessment tool and document review, and qualitative interviews with staff and recently delivered women – to establish the state of hygiene as measured by visual cleanliness and the presence of potential pathogens, and individual and contextual determinants or drivers.

**Results:**

No clear relationship was found between visually assessed cleanliness and the presence of pathogens; findings from qualitative interviews and the facility questionnaire found inadequacies in IPC training for healthcare providers and no formal training at all for ward cleaners. Lack of written policies and protocols, and poor monitoring and supervision also contributed to suboptimal IPC standards.

**Conclusions:**

Visual assessment of cleanliness and hygiene is an inadequate marker for ‘safety’ in terms of the presence of potential pathogens and associated risk of infection. Routine environmental screening of high-risk touch sites using simple microbiology could improve detection and control of pathogens. IPC training for both healthcare providers and ward cleaners represents an important opportunity for quality improvement. This should occur in conjunction with broader systems changes, including the establishment of functioning IPC committees, implementing standard policies and protocols, and improving health management information systems to capture information on maternal and newborn HCAIs.

## Introduction

Improved maternal and newborn health and improved water, sanitation, and hygiene (WASH) are targets of the Sustainable Development Goals and were the subjects of heightened attention as the 2015 Millennium Development Goals deadline approached. However, the synergies between these two targets have been neglected until recently and have tended to focus on WASH in households and the wider community rather than in healthcare institutions (1, 2). However, the state of WASH and infection prevention and control (IPC) in health facilities is slowly gaining attention, as seen in the first global assessment of WASH in health facilities conducted by the World Health Organization (WHO) and UNICEF (3). The report reveals that 38% of facilities surveyed in 54 low- and middle-income countries (LMICs) did not have access to even the most basic WASH services, including soap and water for handwashing.

A long-standing and robust evidence-base shows the links between poor hygiene practices and environment at the time of birth contributing to life-threatening infections in mothers and babies (4). Sepsis remains a leading cause of maternal and neonatal mortality and morbidity, recently estimated to account for up to 10.7% of maternal deaths (5). The Global Burden of Disease Study 2013 further notes that the magnitude of sepsis could be underestimated in countries with high maternal mortality due to difficulties in diagnosis. It also states that the prevention of sepsis should include improved sanitation (6).

The importance of addressing inadequacies in facility-based WASH and IPC is becoming ever more acute given the increasing institutionalisation of deliveries in LMICs, with many countries having reached a tipping point with over 50% of births now taking place in facilities (7). The prospects of this trend leading to health gains for mothers and babies are seriously undermined where health facilities do not have the capacity to cope with the increased demand in terms of trained healthcare workforce and the physical environment, and will inevitably lead to an increase in infection-related morbidity and mortality (8). Few studies exist on the link between increasing institutional deliveries in LMICs, poor IPC, and maternal and newborn infection. Yet what evidence does exist suggests that poor WASH and IPC adversely affect maternal health outcomes through a variety of mechanisms and should be taken into consideration in efforts to improve maternal health (9).

Currently, health facilities are often deemed ‘clean’ based on visual inspection alone (10). One of the few papers on the relationship between cleaning, visual cleanliness and microbiological risk noted that, where wards appeared visibly clean, less than half were safe in terms of the presence of potential pathogens posing an infection risk (11). A further study by Dancer et al. (12) provided evidence of the role of cleaning in healthcare-associated infections (HCAIs). Enhanced cleaning of the intervention ward was associated with a significant reduction in levels of contamination, and a 26.6% reduction in new methicillin-resistant *Staphylococcus aureus* (MRSA) cases compared with the control ward. Despite the importance of facility cleaners and their critical role in maintaining hygiene standards, there is a lack of published literature on these members of the healthcare workforce.

The WASH & CLEAN study was conducted in 2013–2014 by Immpact at the University of Aberdeen, the Indian Institute of Public Health, Gandhinagar (IIPHG), BRAC in Bangladesh, and The Soapbox Collaborative. The study aimed to improve understanding of the determinants of cleaning practices and so inform improvements in the state of cleanliness and safety in maternity units. A suite of tools was developed and applied to a small stratified sample of maternity units in India and Bangladesh to answer the following questions: What are the levels of cleanliness and the determinants (structures), processes, and outcomes of cleaning on the maternity unit? What are the knowledge, attitudes, and practices of stakeholders involved in maintaining cleanliness and their interrelationships? What are the hygiene-related outcomes in terms of visual cleanliness, presence of potential pathogens, and satisfaction of women and healthcare providers (HCPs)?

As this was a novel, exploratory piece of work, a significant amount of data was generated to determine areas where further research is merited. Here we report selected findings that have primary relevance to interventions, highlighting areas with regard to quality improvement as the main, overriding goal of the formative phase of the study. For further information on the remaining findings please contact the corresponding author.

## Methods

### Study design

Ethical approval for the overall study was received from the College Ethics Review Board, University of Aberdeen and The Soapbox Collaborative Ethics Review Board. Ethical approval for the India arm of the study was obtained from the Institutional Ethics Committee of IIPHG; the Government of Gujarat; and management of focus health facilities. Ethical approval for the Bangladesh arm of the study was obtained from the Ethical Review Committee of the James P Grant School of Public Health (ERC ref: 31) at BRAC University. Further permissions were received for public facility inclusion from the Line Director of Medical Education, the Director of Hospital Management Services for the Directorate General of Health Services, and the Ministry of Health and Family Welfare, Government of Bangladesh. Permissions for NGO facility inclusion were received from the Director of the Health, Nutrition and Population Programme of BRAC.

### Pilot and needs assessment

The study tools, described below, were piloted from December 2013 to January 2014 in two maternity units in Gujarat and two in Dhaka Division. Following the pilot, the main formative phase needs assessment was undertaken between February and May 2014 in seven maternity units in Gujarat and eight in Dhaka Division. To ensure a representative sample, maternity units were purposively selected to include public and private facilities, high and low caseloads and facilities offering either Comprehensive Emergency Obstetric Care (CEmOC) or Basic Emergency Obstetric Care (BEmOC).

### Conceptual framework and table of tools

**Fig. 1:**
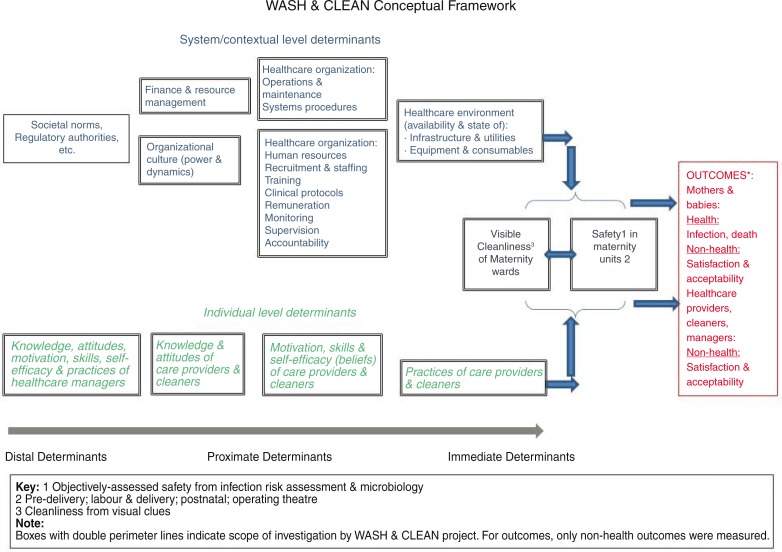
WASH & CLEAN conceptual framework.

Following a review of published and grey literature a conceptual framework was developed, differentiating between three consequences of the state of WASH and IPC in maternity units: 1) safety as captured by microbiological assessment of potential pathogens on high-risk touch surfaces, 2) visual cleanliness, and 3) satisfaction of care users and HCPs. The determinants of these outcomes were differentiated into contextual factors (healthcare infrastructure, standard operating procedures and systems) and individual actors (managers, HCPs, and cleaners) (Fig. 1). Using this framework, existing audit, observational, and survey instruments were adapted to develop a suite of data capture tools: a Walkthrough Checklist, Facility Needs Assessment Tool and Document Capture, and semi-structured interviews with key stakeholders (Table 1).

**Table 1:** WASH & CLEAN table of tools

Tool	Data collection	Data capture topics grouped according to WASH & CLEAN conceptual framework
Walkthrough Checklist	Information collected on the following areas: 1. Determinants of the ‘state of hygiene’ of the maternity ward environment, delivery room environment, and availability and storage of maternity unit cleaning materials 2. Outcomes, that is, the state of hygiene as determined through visual observation, photographs, identification of potential pathogens at selected swab sites, and provider and patient satisfaction	Healthcare environment (systems level determinants) Visible cleanliness (outcome) Presence of potential pathogens (outcome)
Facility Needs Assessment Tool	Questionnaire administered in an interview format with the head nurse, or equivalent, of the maternity unit	Healthcare organisation, system, and operations (systems level determinants) (Human) resources (systems level determinants) IPC and healthcare practices (individual and systems level determinants)
Document Capture	Checklist of policies and protocols relevant to IPC	Healthcare system and operations (systems level determinants)
Semi-structured interviews	Semi-structured interviews with management, healthcare providers, cleaners, and recently delivered women	Motivation, skills, and self-efficacy (individual level determinants) IPC and healthcare practices (individual and systems level determinants) Healthcare organisation, system, and operations (systems level determinants) (Human) resources (systems level determinants) Finance and resource management (systems level determinants) Providers and managers’ satisfaction (outcomes) Women's satisfaction (outcomes)

### WASH & CLEAN tools

#### Walkthrough Checklist

The Walkthrough Checklist involved recording standard aspects of IPC at specific moments and locations while passing (walking) through the maternity unit. Walkthrough Checklist data were captured through three modalities: 1) completion of an observational checklist, 2) collecting of swab samples from high-risk touch sites, and 3) taking photographs of swab sites as well as relevant infrastructure and equipment, such as delivery beds and handwashing stations.

Checklist questions related to the determinants of the state of hygiene, such as ‘Is water currently available for hand washing in the delivery room?’, and to the state of hygiene, such as ‘Are water points for hand washing in the delivery room visibly clean? Are they free from debris?’. Responses to the questions were pooled and used to create summary percentage scores. The state of hygiene determinants score (SOH-D score) and the visual state of hygiene score (SOH-V score) were then grouped according to quartiles, with a score of 75% or more labelled ‘very good’, 50–74% ‘good’, 25–49% ‘moderate’, and 0–24% ‘poor’. This approach to scoring and the use of quartiles is common practice in ‘improvement science’ – providing benchmarks of performance, aiding priority setting, and providing markers for tracking progress (13). Further details on the tools are available in the WASH & CLEAN toolkit at www.soapboxcollaborative.org.

During the walkthrough process, swab samples were taken at up to 30 designated sites per facility in the maternity ward, delivery room, and cleaners’ storeroom to gain an objective measure of infection risk. Samples were analysed in local laboratories based at teaching hospitals in Gujarat and Dhaka. *Staphylococcus aureus* (including coagulase-negative staphylococci) is one of the most common causes of HCAIs (14), causing both skin and soft tissue infections, and invasive infections such as septicaemia, and was thus the primary pathogen of interest. The presence of additional potential pathogens (*Streptococcus*, *Klebsiella*, and *Pseudomonas*) was also reported. The additional pathogens, while not of primary interest, indicate poor environmental hygiene and lack of effective cleaning. Non-pathogenic organisms such as *Bacillus subtilis* were also reported and, while not posing a high risk to patients, are indicative of poor cleaning practices (15). Photographs of swab sites were taken as a means of verifying the reported visual assessments made when conducting the checklist.

To minimise the Hawthorne effect, efforts were made to apply the Walkthrough Checklist before the remaining five tools. Health facility management was requested to avoid disclosing the precise time and date of data collection to practitioners. As part of the approval process, management was aware of the data to be captured; however, this was not conveyed to the wards where data capture took place.

#### Facility Needs Assessment Tool and Document Capture

The Facility Needs Assessment Tool (questionnaire) and Document Capture gathered information on infrastructure and utilities; training; IPC resource availability, policies, and protocols; and routine practices. Data collectors completed the questionnaire during an interview with the senior nurse, or equivalent, from the maternity unit.

#### Semi-structured interviews with stakeholders

Semi-structured interviews were conducted with a range of stakeholders (five members of management, 21 HCPs, 19 ward cleaners, and 25 women who had received maternity care from the participating facilities). Interviews used a technique called photo-elicitation whereby photo-prompts are used to generate discussion and insights rarely gained through direct questioning (16). Photo-prompts in this study included examples of delivery rooms and toilets. Qualitative analysis took a framework approach based on the conceptual framework with the aim of exploring views and perceptions of the determinants of hygiene and the state of hygiene in the maternity unit. The framework approach was selected as an effective and flexible approach to qualitative data analysis, particularly in mixed method studies (17).

Participants provided verbal consent to participate in the study. Due to the fact that data collection with participants took the form of recorded interviews, recorded verbal consent was deemed sufficient. Consent procedures received ethical approval prior to study commencement.

### Data analysis

Categorical data from the Walkthrough Checklist and Facility Needs Assessment Tool were entered into an SPSS database at IIPHG and BRAC. Prior to analysis, the data were checked for internal consistency. Descriptive statistics were produced using SPSS 20. ATLAS.ti was used to undertake thematic analysis of the qualitative interview data. Interviews were conducted, transcribed, and analysed in-country in the local language. Results of the transcript analysis were translated into English and back-translated.

From the swab samples, species identification was conducted using Gram staining and standard biochemical tests.

## Results

Due to their relevance to interventions and highlighting areas with regard to quality improvement, findings related to the Walkthrough Checklist (microbiology, SOH-V score, and SOH-D score), and training, monitoring, and document availability have been selected for reporting. Differences between facilities with regard to organisational context (private for profit, government, private not for profit, etc.) and caseload did not show any consistent patterns; while differences existed, they did not lie reliably in one direction. Thus, the results are presented only by country and obstetric functionality, that is, BEmOC or CEmOC.

### Visual state of hygiene (SOH-V) and Determinants of the state of hygiene (SOH-D)

The overall summary score for the visually assessed state of hygiene (SOH-V score) across all maternity units in Gujarat was close to 50%, indicating a ‘good’ state of visible cleanliness. There was comparatively little variability, with the exception of one CEmOC facility which scored 96%. Scores for facilities in Dhaka Division ranged from 35 to 100%, indicating a ‘moderate’ to ‘very good’ state of hygiene according to visual inspection.

The average summary score for the determinants of the state of hygiene (SOH-D score) across all maternity units in Gujarat was 60%, indicating a ‘good’ presence of key determinants or requirements for maintaining IPC; there was very little variability between facilities with one facility rating ‘very good’ and the remainder ‘good’. Scores for Dhaka Division varied to a greater degree, ranging from 38 to 100% indicating a ‘moderate’, ‘good’, or ‘very good’ presence of the key determinants or requirements for maintaining hygiene standards.

**Fig. 2:**
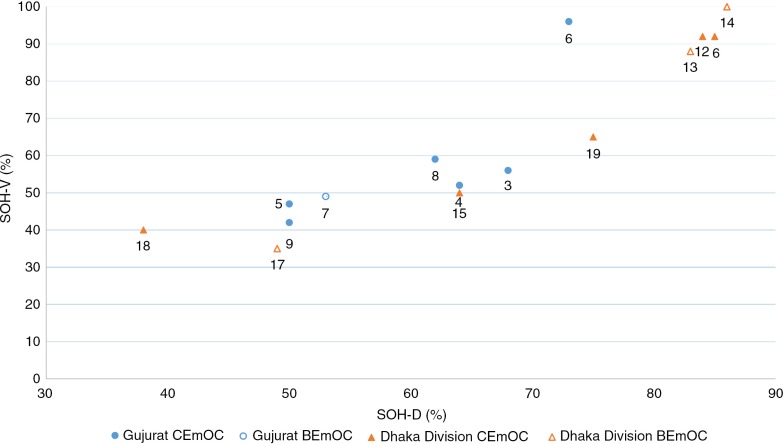
Relationship between scores for visually assessed state of hygiene (SOH-V) and determinants of the state of hygiene (SOH-D) by country, facility number, and obstetric functionality.

Figure 2 provides a simple illustration of the relationship between the SOH-V score and the SOH-D score. Reassuringly, there is a clear and predictable positive association, with high visual states of hygiene matching high scores for the overall provision of determining factors.

Differences between the positive responses for the sub-components of the Walkthrough Checklist were examined. Albeit in some cases the numbers were small, the results do show some priorities for improvement indicating differences between facilities in terms of specific areas of IPC, such as waste storage and disposal, and maternity ward toilets (Tables 2 and 3 and Supplementary File).

**Table 2:** Walkthrough Checklist section by individual facilities^a^

Walkthrough Checklist section	Gujarat determinants	Gujarat outcomes	Dhaka Division determinants	Dhaka Division outcomes
Maternity ward general area and handwashing	0.176	**<0.001**	**<0.001**	**<0.001**
Maternity ward beds	0.995	**0.034**	0.881	**0.012**
Maternity ward toilets	0.796	**0.046**	0.096	NA
Delivery unit general area and handwashing	0.290	**0.014**	**<0.001**	**0.001**
Delivery unit waste storage and disposal	**<0.001**	0.664	**<0.001**	NA
Cleaning materials and linen	**0.020**	**0.007**	**0.031**	**0.001**

a Fisher's exact test applied (bold denotes statistically significant *p*-value at <0.05% level).

**Table 3:** Walkthrough Checklist section by facilities grouped according to obstetric functionality^a^ (CEmOC/BEmOC)

Walkthrough Checklist section	Gujarat determinants	Gujarat outcomes	Dhaka Division determinants	Dhaka Division outcomes
Maternity ward general area and handwashing	0.177 (F)	0.176 (F)	0.081	0.080
Maternity ward beds	0.572 (F)	0.373	0.672	0.874
Maternity ward toilets	0.281 (F)	0.471 (F)	0.203	0.136
Delivery unit general area and handwashing	0.551	0.370	0.808	0.324
Delivery unit waste storage and disposal	0.625	0.396 (F)	0.079	0.790
Cleaning materials and linen	0.896	0.584	0.401	0.434

a Fisher's exact test (F) or Chi-square test applied. CEmOC, Comprehensive Emergency Obstetric Care; BEmOC, Basic Emergency Obstetric Care.

### Microbiology

Laboratory analysis proceeded differently in Dhaka and Gujarat. Results for Dhaka Division only are reported due to the robustness of *S. aureus* identification and characterisation. In Dhaka Division, colony counts were reported as per the protocol. In Gujarat, only the presence/absence of pathogens was reported, which makes a direct comparison between the countries difficult.

In Dhaka Division, *S. aureus* was most commonly found on delivery room door handles and maternity ward bed(s) at the approximate location of patients' hands and feet (NB: maternity ward beds are for antenatal, early labouring or postnatal cases; delivery beds are for women in advanced labour and is where the baby is delivered). While *S. aureus* was the pathogen of interest, the laboratories reported the presence of additional potential pathogens that would indicate poor environmental hygiene and lack of effective cleaning. Potential pathogens including *Klebsiella* and *Pseudomonas* were found across all facilities in both countries. Non-pathogenic organisms such as *Bacillus subtillis* were also found at the sample sites, and while not posing a high risk to patients, these organisms are indicative of poor cleaning practices.

**Fig. 3:**
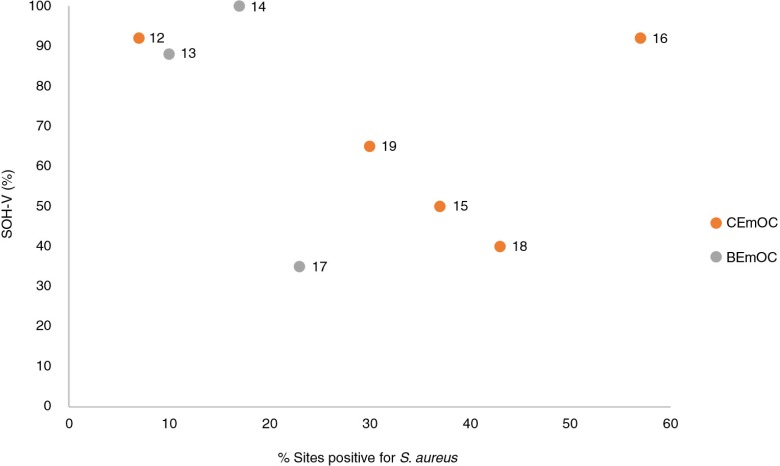
SOH-V score by % sites testing positive for *S. aureus* (Dhaka Division). NB: Numbers 12–19 refer to facility identification codes.

Figure 3 shows the distribution of facilities in Dhaka Division according to their overall SOH-V score and the proportion of sites testing positive for *S. aureus*. It is noteworthy that despite scoring ‘very good’ in terms of the visually assessed state of hygiene (>75%), 57% of swab samples taken at facility 16 tested positive for *S. aureus*.

### Training and monitoring

Interviews were conducted with key stakeholders exploring views and perceptions of the determinants of hygiene and the state of hygiene in the maternity unit. As stated above, due to their relevance in terms of intervention, only selected findings pertaining to training and monitoring are reported here.

Training was a key area of need as raised repeatedly throughout the interviews with stakeholders. Findings on training provision, as captured by the Facility Needs Assessment Tool, are presented in Table 4. Training was notably absent in the majority of facilities in Gujarat and in the two facilities with the lowest SOH-V scores in Dhaka Division.

**Table 4:** Infection prevention and control training

Facility No. (Gujarat facilities 3–9; Dhaka Division facilities 12–19)	Obstetric functionality	Overall state of hygiene determinants score (SOH-D)	Overall visual state of hygiene score (SOH-V)	Any training in IPC conducted in the last year?	Orientation programme with information on IPC for new HCPs?	Training programme in IPC for all HCPs?	Training programme in IPC for non-clinical staff (ward cleaners, maintenance staff, etc.)?
3	CEmOC	68	56	No	No	No	No
4	CEmOC	64	52	No	No	No	No
5	CEmOC	50	47	No	No	No	No
6	CEmOC	73	96	No	No	No	No
7	BEmOC	53	49	Yes	No	Yes^a^	No
8	CEmOC	62	59	No	No	No	No
9	CEmOC	50	42	No	No	No	No
12	CEmOC	83	92	Yes	Yes	No	Yes^b^
13	BEmOC	83	88	Yes	Yes	Yes	Yes
14	BEmOC	86	100	Yes	Yes	Yes	Yes
15	CEmOC	64	50	No	No	No	Yes^b^
16	CEmOC	85	92	Yes	Yes	Yes	Yes^b^
17	BEmOC	49	35	No	No	No	No
18	CEmOC	38	40	No	No	No	No
19	CEmOC	75	65	Yes	No	No	Yes^b^

a Two HCPs trained;

b included ward cleaners in training.

While the awareness of the importance of IPC was good among all stakeholders, interviewees in Gujarat reported that inadequate training for HCPs and no systematic training for cleaners were major bottlenecks in all facilities. Managers noted that currently training is ‘suboptimal’ and discussed the lack of knowledge and awareness of Class 4 (cleaning) staff:

If Class 4 [cleaning staff] is given education and various training regarding infection, and what are the problems to the patient due to infection, and what is the effect of infection on maternal and infant death, … in their local language, so it will be a good improvement. Manager (CEmOC, Gujarat)

Facilities with high SOH-V scores in Dhaka Division reported providing IPC training for new staff, non-medical staff, and existing staff (with the exception of facility 12). Yet interview results revealed inconsistencies in the reported availability of training and actual delivery of training. Nine of the 18 HCPs interviewed had received training on hand washing only, while just five of the 22 cleaners interviewed had received orientation training on hand washing and overall cleanliness. Most training appeared to be informal and/or ‘on-the-job’:

We received only one day orientation training on IPC here … but it was not very formal.

HCP (BEmOC, Dhaka Division)

I did not get any training on IPC here. I have enriched myself, learning by doing.

Cleaner (CEmOC, Dhaka Division)

One manager however appeared contradictory, stating that while training should be provided, it is not necessary to formally train all staff due to on-the-job training.

Training is suboptimal. Because it is not necessary to have all trained staff. Staff learn when they work and learn with seniority. In any set up, it is better to give training. Manager (CEmOC, Gujarat)

According to a clinic in-charge and the managers of two facilities, arranging training for clinic assistants and cleaners was a neglected issue:

Management asked us to arrange one day IPC training for clinic assistants and cleaners. I do not think we are knowledgeable enough to provide training to others. We organised a training session and delivered information to cleaners and clinic assistants, whatever we have learned from IPC trainers. Actually I am not satisfied with that session. Manager (CEmOC, Dhaka Division)

I am not the authority to organise training for staffs … if management does not take any initiative … we have nothing to do. We only instructed cleaners verbally about IPC procedure. Manager (CEmOC, Dhaka Division)

The interview results also suggested that overall monitoring of IPC was generally poor across the facilities, reflecting the lack of formal committees charged with this role as captured by the Facility Needs Assessment Tool. Several stakeholders discussed improvements in practices that could result from adequate supervision and accountability (particularly in relation to permanent staff):

It is also important to know whether implementation is done or not as per the training. Timely audit and supervision should be done to evaluate it. HCP (CEmOC, Gujarat)

Personal responsibility for monitoring was also acknowledged.

Actually it is not possible to monitor all IPC related activities by a person. Everyone should be responsible for it. HCP (CEmOC, Dhaka Division)

A lack of documentation on sepsis cases was noted across facilities, reflecting poor health management information systems. Some information was captured by non-public facilities in Bangladesh, but this was the exception to the rule. In general, presence of policies and protocols was poor. Only half of the facilities in Dhaka Division had selected written policies and protocols related to IPC, and while documents reportedly existed in the remaining facilities, only one or two were available. None of the facilities in Gujarat reported policy documents or written protocols on cleaning and IPC, although some reported ‘undocumented’ protocols.

## Discussion

Much of the global focus on preventing HCAIs has concentrated on hand hygiene (18). This is an essential intervention but needs to be accompanied by a hygienic physical environment in order to break the transmission chain of infection (19). This is particularly important for clinical areas caring for patients at higher risk, and with vulnerable sites, such as delivery beds. A crucial enabling factor in the physical environment is the basic requirement for water and sanitation – a requirement which the combined results of our study and other assessments in low-income settings (e.g. GLAAS and WHO & UNICEF) show to be lacking (3, 20). This gap also represents a major opportunity for improvement.

While differences exist between the two participating countries, for example, the Facility Needs Assessment Tool results in terms of the reported provision of training in IPC, here we focus on common themes emerging from the results in relation to microbiology, visual cleanliness and the determinants of the state of hygiene, training and monitoring, and policies and protocols.

The reliance on visual cleanliness as a proxy for ‘safety’ is currently widespread; national and international guidelines often use visual cleanliness and frequency of cleaning as indicators of the extent to which IPC standards are met [e.g. the Centres for Disease Control and Prevention, and the UK National Health System (21, 22)]. In this study, there does not appear to be a clear relationship between the presence of the clinically important pathogen *S. aureus* at key sites and visually assessed cleanliness, which may suggest the need for routine monitoring of hygiene safety going beyond subjective observation.

A non-trivial proportion of specific potential pathogens, such as *S. aureus*, exist in the healthcare environment due to normal human carriage from the community (13, 23); thus, it is not unusual for a proportion of high-risk sites in clinical settings to test positive for *S. aureus*. Nevertheless, an interesting finding of this study was wide differences in the presence of *S. aureus* between facilities and between swab sites within the same facility, which may suggest inconsistent implementation of IPC standards across facilities. Whether a potential pathogen causes infection depends on many factors, including the vulnerability of the host (24). It is well accepted that patients on maternity units – both mothers and babies – face particular risks owing to the physiological processes of birth, such as cutting the umbilical cord, perineal tears, or caesarean section wounds. All CEmOC facilities face particular challenges regarding cross-infection, particularly where space is constrained and high-risk cases are managed post-operatively in the same beds and clinical area as uncomplicated cases.

Based on our results and the current literature, there is a strong case to argue that visual assessment of cleanliness on maternity units alone is an inadequate basis on which to determine safety in terms of the presence of potential pathogens. Yet in many low-income healthcare settings overall laboratory capacity is often weak, helping to explain the limited application of environmental screening and the heavy reliance on visible cleanliness alone. While the need to strengthen medical laboratories is widely acknowledged (25), options for simplifying environmental microbiology techniques for swabbing hard surfaces, and culturing and reading plates, along with training technicians, could have significant benefits for routine monitoring and supervision of hygiene on maternity units at many levels of the health system, and not just major hospitals.

In the WASH & CLEAN study, the findings from the application of the Walkthrough Checklist show a clear association between the composite scores for the visually assessed state of hygiene (SOH-V score) and for the determinants of the state of hygiene (SOH-D score). However, the results of the Facility Needs Assessment Tool suggest a more complicated picture as regards the reported availability of resources crucial to IPC (not reported here). Some facilities lacking such resources still reported that a high proportion of IPC practices were performed routinely, perhaps suggesting the difficulty of declaring non-compliance. The findings also point to the influence of staff shortages on IPC practices and of training and staff motivation enabling good performance even in the face of resource shortage. The lack of training provided to cleaners and HCPs across facilities as a whole in the WASH & CLEAN study is an area warranting future improvement, including the development of novel methods of engagement suitable for personnel with minimal formal education.

Common bottlenecks to IPC included a lack of policies, protocols, monitoring, supervision, and accountability. Gaps were also identified during the study that warrant implementation research, such as establishing and sustaining effective IPC committees, routine supportive monitoring and supervision of cleaning staff, and strengthening the use of simple audit cycles within a culture of hygiene safety.

The reported absence of the implementation of formal, systematic training for cleaners was universal across participating facilities, reflecting a general undervaluing of this cadre. This was also apparent from the interviews where ward cleaners' poor remuneration and benefits, and a lack of contractual security in many instances, were reported. The lack of training for cleaners and HCPs across facilities is an area warranting future improvement. The experience from our study points to the potential to develop and test a bundle of interventions around cleaning practices and cleaners, taking into account the context of cleaning, skills mix, and educational background of cleaning staff in LMICs. Training must also address socially and culturally specific drivers and beliefs relating to cleanliness and hygiene that are not amenable to influence solely by standard IPC policies and processes. Development of such training should take into account successful community-based interventions around behaviour change in this area, such as Curtis et al. (26, 27).

The experience of our study points to the value of mixed methods and sources of data to monitor the state of hygiene on maternity units. These include observational and microbiology techniques and practical mechanisms to triangulate findings and handle data by the facilities themselves.

In terms of limitations, the study did not capture data on maternal or newborn sepsis occurring among deliveries in the participating facilities, and thus the study findings cannot be linked directly to health outcomes. Although non-public facilities in Dhaka Division had an established health management information system, the lack of available routine data on the prevalence and risk factors for sepsis for mothers and newborns was notable in the remaining facilities. The second limitation, common to other studies using observational methods, was the difficulty of avoiding the Hawthorne effect. Although we sought to minimise inter-rater variability as far as possible, inevitably data collectors’ perceptions would have influenced reporting of visible cleanliness. However, the researchers who analysed the data and interpreted the findings were independent from the data capture processes. There were challenges in conducting the environmental microbiology analysis, which is not widely undertaken in such study settings, and detailed quality assurance of the laboratory results was not undertaken. Yet this study has provided an indication of the potential to use environmental microbiology as an objective assessment of hygiene risk to complement visual inspection. In terms of the qualitative data collection, there is a general difficulty interviewing health workers in busy facilities, which may have had an impact on the quality of data captured. Moreover, the need to manage the withdrawal of staff from duties to participate in interviews resulted in facility managers selecting staff for interview which may have inadvertently introduced selection biases in the representativeness of interviewees.

A further limitation was the use of simple ‘yes/no’ responses to questions in the Walkthrough Checklist rather than a scale to capture degrees of visual cleanliness. However, this practice was consistent with the generic tools from which the WASH & CLEAN study instruments were developed and avoided creating an overly complex data set. By pooling variables in the checklist to create an overall score, the analysis is also potentially masking areas for improvement and/or of existing good practice which are important for actions at a facility level; the study did not differentially weigh the variables used in the summary scores, as this was not recommended by the generic tools. However, further analysis will investigate simple means of weighting.

Future research into the pathways of infection is needed. What evidence exists suggests a link between environmental hygiene and risk of infection, yet little research has been conducted in LMICs, and even less around risks posed specifically to mothers and newborns. The lack of routine data on the prevalence and risk factors for maternal and newborn sepsis was notable in the participating facilities. To generate political will and secure buy-in at all facility levels, there is a need to find practical mechanisms for the healthcare workforce to appreciate the consequences of poor hygiene practices for the patients they care for, and to provide further evidence of the link between environmental hygiene and infection. Research is needed not only to strengthen health information systems, but also assess the impact of direct feedback to staff on HCAIs in terms of changing their hygiene behaviour.

Publication of the recent WHO and UNICEF report (3) on WASH in health facilities and increasing global attention on the importance of improving WASH and IPC in health facilities provides us with a major opportunity and an obligation to act. Our study demonstrates the importance of addressing the multifaceted nature of WASH and IPC on maternity units. Starting with a low baseline – absence of dedicated training for cleaners, lack of appropriate monitoring and supervision, etc. – means there is considerable room for improvement. Ultimately, such a focused intervention should be integrated with other systems improvements for IPC in health facilities. Such actions will not only benefit mothers, newborns, and staff on maternity units but also those seeking or providing care in other important clinical settings.

## Supplementary Material

Hygiene on maternity units: lessons from a needs assessment in Bangladesh and India

## Paper context

Health system standards of infection prevention and control, and water, sanitation, and hygiene in low- and middle-income countries are often neglected but essential to quality care. Needs assessments with novel tools were conducted in maternity units in India and Bangladesh. Among the findings, an unclear relationship was found between visually assessed cleanliness and the presence of pathogens, and no formal training for ward cleaners. Assessments should be scaled up and action taken to address inadequacies.
