# Supplementary material for: Hygiene on maternity units: lessons from a needs assessment in Bangladesh and India
Source: Glob Health Action. 2016 Dec 12;9:10.3402/gha.v9.32541. doi: 10.3402/gha.v9.32541 (PMC5155114; doi:10.3402/gha.v9.32541)
Supplement: Hygiene on maternity units: lessons from a needs assessment in Bangladesh and India [file GHA-9-32541-s001.pdf]

SUPPLEMENTARY MATERIAL: Walkthrough Checklist Results

Table 1: Gujarat - Individual facility determinants & outcomes scores by Walkthrough Checklist section

Gujarat Determinants Score - n (%)								
	Facility Number							
**Walkthrough Checklist Section†**	**3**	**4**	**5**	**6**	**7**	**8**	**9**	**Average**
**MW General Area & Handwashing**	2 (33.3)	8 (61.5)	8 (61.5)	9 (69.2)	1 (20.0)	1 (20.0)	1 (20.0)	4 (40.8)
**MW Beds**	3 (75.0)	2 (50.0)	3 (60.0)	4 (80.0)	3 (75.0)	3 (60.0)	3 (60.0)	3 (65.7)
**MW Toilets**	2 (50.0)	4 (57.1)	4 (57.1)	5 (71.4)	1 (25.0)	2 (50.0)	1 (25.0)	3 (51.4)
**DU General Area & Handwashing**	19 (79.2)	15 (65.2)	12 (54.6)	16 (72.7)	14 (73.7)	10 (47.6)	13 (59.0)	14 (64.7)
**DU Waste Story & Disposal**	10 (90.0)	12 (92.3)	2 (18.2)	2 (20.0)	7 (63.6)	6 (54.6)	5 (50.0)	6 (57.1)
**Materials & Linen**	12 (52.2)	12 (52.2)	10 (43.5)	21 (91.3)	13 (56.5)	12 (52.2)	13 (56.5)	13 (57.8)
**Gujarat Outcomes Score - n (%)**								
	Facility Number							
**Walkthrough Checklist Section**	**3**	**4**	**5**	**6**	**7**	**8**	**9**	**Average**
**MW General Area & Handwashing**	3 (75.0)	5 (45.5)	6 (54.6)	13 (100.0)	6 (75.0)	0 (0.0)	2 (33.3)	35 (59.3)
**MW Beds**	3 (42.9)	2 (28.6)	7 (77.8)	9 (100.0)	4 (57.1)	4 (44.4)	4 (44.4)	33 (57.9)
**MW Toilets**	1 (25.0)	4 (50.0)	2 (25.0)	7 (87.5)	1 (25.0)	0 (0.0)	1 (25.0)	16 (40.0)
**DU General Area & Handwashing**	10 (55.6)	12 (80.0)	10 (58.8)	16 (100.0)	9 (64.3)	8 (50.0)	9 (56.3)	74 (66.1)
**DU Waste Story & Disposal**	2 (100.0)	2 (100.0)	0 (0.0)	1 (50.0)	1 (50.0)	1 (50.0)	2 (100.0)	9 (64.3)
**Materials & Linen**	3 (50.0)	3 (37.5)	1 (10.0)	8 (100.0)	5 (62.5)	3 (37.5)	3 (37.5)	26 (46.4)

†MW – Maternity Ward; DU – Delivery Unit

Table 2: Dhaka Division - Individual facility determinants & outcomes scores by Walkthrough Checklist section

**Dhaka Division Determinants Score - n (%)**									
	**Facility Number**								
**Walkthrough Checklist Section†**	**12**	**13**	**14**	**15**	**16**	**17**	**18**	**19**	**Average**
**MW General Area & Handwashing**	8 (66.7)	7 (58.3)	8 (66.7)	1 (8.3)	14 (87.5)	12 (92.3)	12 (92.3)	5 (41.7)	67 (65.7)
**MW Beds**	5 (100.0)	4 (80.0)	5 (100.0)	3 (60.0)	4 (80.0)	4 (80.0)	4 (80.0)	3 (75.0)	32 (82.1)
**MW Toilets**	5 (71.4)	3 (37.5)	4 (57.1)	0 (0.0)	4 (57.1)	5 (71.4)	5 (71.4)	3 (42.9)	29 (50.9)
**DU General Area & Handwashing**	19 (82.6)	18 (78.3)	21 (87.5)	12 (52.2)	24 (100.0)	22 (91.7)	22 (91.7)	12 (52.2)	150 (79.8)
**DU Waste Story & Disposal**	11 (84.6)	8 (61.5)	13 (100.0)	2 (15.4)	7 (53.9)	13 (100.0)	13 (100.0)	5 (38.5)	72 (69.3)
**Cleaning Materials & Linen**	22 (88.0)	14 (56.0)	21 (84.0)	14 (56.0)	14 (56.0)	15 (60.0)	18 (72.0)	13 (54.2)	131 (65.8)
**Dhaka Division Outcomes Score - n (%)**									
	**Facility Number**								
**Walkthrough Checklist Section**	**12**	**13**	**14**	**15**	**16**	**17**	**18**	**19**	**Average**
**MW General Area & Handwashing**	13 (81.3)	6 (46.2)	12 (75.0)	3 (21.4)	9 (56.3)	14 (87.5)	16 (100.0)	5 (31.3)	78 (63.4)
**MW Beds**	9 (100.0)	6 (66.7)	9 (100.0)	5 (55.6)	5 (55.6)	5 (83.3)	6 (100.0)	3 (42.9)	48 (75.0)
**MW Toilets**	8 (100.0)	0 (0.0)	8 (100.0)	0 (0.0)	3(37.5)	8 (100.0)	8 (100.0	0 (0.0)	35 (54.7)
**DU General Area & Handwashing**	16 (88.9)	14 (77.8)	17 (94.4)	12 (70.6)	17 (94.4)	14 (93.3)	15 (100.0)	8 (47.1)	113 (83.1)
**DU Waste Story & Disposal**	2 (100.0)	0 (0.0)	2 (100.0)	0 (0.0)	2 (100.0)	2 (100.0)	2 (100.0)	0 (0.0)	10 (62.5)
**Cleaning Materials & Linen**	10 (100.0)	4 (40.0)	10 (100.0)	4 (40.0)	5 (50.0)	7 (70.0)	7 (100.0)	4 (57.1)	51 (68.9)

†MW – Maternity Ward; DU – Delivery Unit

Table 3: Gujarat - Facility determinants & outcomes scores grouped according to obstetric functionality† by Walkthrough Checklist section

**Gujarat Determinants Score - n (%)**			
**Walkthrough Checklist Section‡**	**CEmOC (N, %)**	**BEmOC (N, %)**	**Total (N, %)**
**MW General Area & Handwashing**	29 (52.7)	1 (20.0)	30 (50.0)
**MW Beds**	18 (66.7)	3 (60.0)	21 (65.6)
**MW Toilets**	18 (54.6)	1 (25.0)	19 (51.4)
**DU General Area & Handwashing**	86 (65.6)	13 (59.1)	99 (64.7)
**DU Waste Story & Disposal**	39 (58.2)	5 (50.0)	44 (57.1)
**Materials & Linen**	80 (58.0)	13 (56.5)	93 (57.8)
**Gujarat Outcomes Score - n (%)**			
**Walkthrough Checklist Section**	**CEmOC (N, %)**	**BEmOC (N, %)**	**Total (N, %)**
**MW General Area & Handwashing**	33 (62.3)	2 (33.3)	35 (59.3)
**MW Beds**	29 (60.4)	4 (44.4)	33 (57.9)
**MW Toilets**	15 (41.7)	1 (25.0)	16 (40.0)
**DU General Area & Handwashing**	65 (67.7)	9 (56.3)	74 (66.1)
**DU Waste Story & Disposal**	7 (58.3)	2 (100.0)	9 (64.3)
**Materials & Linen**	23 (47.9)	3 (37.5)	26 (46.4)

† Comprehensive Emergency Obstetric Care (CEmOC)/Basic Emergency Obstetric Care (BEmOC)
‡DU – Delivery Unit; MW – Maternity Ward

Table 4: Dhaka Division - Facility determinants & outcomes scores grouped according to obstetric functionality† by Walkthrough Checklist section

**Dhaka Division Determinants Score - n (%)**			
**Walkthrough Checklist Section‡**	**CEmOC (N, %)**	**BEmOC (N, %)**	**Total (N, %)**
**MW General Area & Handwashing**	38 (59.4)	29 (76.3)	67 (65.7)
**MW Beds**	21 (84.0)	11 (78.6)	32 (82.1)
**MW Toilets**	16 (44.4)	13 (61.9)	29 (50.9)
**DU General Area & Handwashing**	94 (80.3)	56 (78.9)	150 (79.8)
**DU Waste Story & Disposal**	41 (63.1)	5 (50.0)	72 (69.2)
**Cleaning Materials & Linen**	85 (68.0)	46 (62.2)	131 (65.8)
**Dhaka Division Outcomes Score - n (%)**			
**Walkthrough Checklist Section**	**CEmOC (N, %)**	**BEmOC (N, %)**	**Total (N, %)**
**MW General Area & Handwashing**	43 (57.3)	35 (72.9)	78 (63.4)
**MW Beds**	34 (75.6)	14 (73.7)	48(75.0)
**MW Toilets**	19 (47.5)	16 (66.7)	35 (54.7)
**DU General Area & Handwashing**	76 (85.4)	37 (78.7)	113 (83.1)
**DU Waste Story & Disposal**	6 (60.0)	4 (66.7)	10 (62.5)
**Cleaning Materials & Linen**	33 (66.0)	18 (75.0)	51 (68.9)

† Comprehensive Emergency Obstetric Care (CEmOC)/Basic Emergency Obstetric Care (BEmOC)
‡DU – Delivery Unit; MW – Maternity Ward
